# Potential and Prophylactic Use of Plants Containing Saponin-Type Compounds as Antibiofilm Agents against Respiratory Tract Infections

**DOI:** 10.1155/2021/6814215

**Published:** 2021-07-23

**Authors:** I. Irem Tatli Cankaya, E. Inci Somuncuoglu

**Affiliations:** ^1^Hacettepe University, Faculty of Pharmacy, Department of Pharmaceutical Botany, Sıhhiye, Ankara, Turkey; ^2^Department of Health Services, Ministry of Health, Ankara, Turkey

## Abstract

Epidemic diseases have been observed in every period of human history, and the treatment process has taken time. Causative microorganisms reproduce as biofilm and contribute to the emergence of various infectious diseases. The process that starts with respiratory disorders causes serious lung infections due to bacteria and viruses that accumulate and multiply. The biofilms are difficult to eliminate and show increased resistance to available antimicrobial agents. There is a need to identify and develop potential resources used in treatment. The search for novel biological agents from plants is gaining popularity due to the high abundance, accessibility with consequent lower cost for discovery, and lesser side effects and toxicity. Saponins found in some plants can be alternative to antibiotics, with antimicrobial activities. This review focused on the potency of saponin-containing plants with antimicrobial properties as antibiofilm agents against these infections. For this purpose, keywords were scanned in Web of Science, Scopus, and Google academics databases, and the related literature was compiled. Approximately, 25 plant taxa belonging to 18 families traditionally used in the treatment of respiratory diseases are listed. These taxa mostly belong to Fabaceae, Asteraceae, Apiaceae, and Asparagaceae families, respectively. Most of these taxa have antibacterial, antifungal, antitussive, and anti-inflammatory activities. Especially, plants with antibiofilm activity that can be effective against many microorganisms are compiled in this study. These plants can prevent or treat upper respiratory tract diseases caused by bacteria due to the phytochemicals they contain, especially saponins.

## 1. Introduction

Microbial biofilms can be defined as surface-bound microorganism communities that form on living and inanimate solid surfaces and grow embedded in a matrix of extracellular polymeric materials. They are imagined as a significant virulence factor causing permanent chronic and repetitive infections; they are extremely durable to antimicrobials/antibiotics and host immune defenses. This situation seriously complicates treatment options. Approximately 75% of bacterial infections contain biofilms maintained by an extracellular matrix. Various reasons, such as limited diffusion of antibiotics into the biofilm matrix, expression of multidrug efflux pumps, type IV secretion systems, reduced permeability, and the effect of antibiotic modifying enzymes cause biofilm resistance [1–4].

In recent studies, it has been dealt with *Staphylococcus aureus* and *S*. *epidermidis* biofilms more specifically. Although they are often described as common species and permanent colonizers of human oromucosal membranes, they may often be opportunistic pathogens compared to premature newborns and immunocompromised patients. They are among the most extensive sources of infection in permanent medical implanted instruments, such as catheters. They can cause serious chronic infections [5, 6]. In addition, *Pseudomonas aeruginosa* is Gram-negative bacteria recognized as a lethal and resistant bacterium that can create critical infections in immunocompromised patients and is also one of the main causes of nosocomial infections, septic shock, bronchopneumonia, and wound infections. When the bacteria form a biofilm, it is highly rough to devastate the infection. The bacteria are then more resistant to antibiotic treatment and the host immune response. This adaptability may have provided multiple drug resistance properties [7, 8].

*Candida albicans* is the best known and the most effective opportunistic fungal pathogen of humans and animals. It creates an extensive microflora in the gastrointestinal and genitourinary tracts of 70% of people. However, in some cases, *C*. *albicans* can be pathogenic for patients with critical illness, immune deficiency, and even healthy people [9, 10]. The incidence of candidiasis has increased in recent years, especially in immunocompromised patients. Reports indicate that *Candida*'s antifungal drug resistance is possible [11].

Prevention of biofilm is imagined as the main drug target in the treatment of a variety of bacterial and fungal infections, and the pharmacological improvement of these drugs is currently being widely investigated.

Antibiotic-resistant bacteria cannot be controlled or destroyed by antibiotics. They can survive or even reproduce in the presence of an antibiotic. The most critical concern with antibiotic resistance is that some bacteria have resistant to almost all antibiotics that are readily available. As a precaution, national and international principles and guidelines have been developed for the use of antibiotics and administration based on clinical evidence. Nevertheless, the number of antibiotics used and effective in this sense is very low. Antibiotics such as rifampin are widely used together with other antibiotics to cure *S*. *aureus* infections and seem promising in the treatment of infections; however, the data that support this application are limited and more precise data are not available [12].

Recently, various green nonlethal strategies for biofilm control have been improved. A promising alternative is to investigate naturally occurring plant-derived compounds that can block biofilm formation. Historically, plant extracts and bioactive components have been precious sources of natural products that play a central role in avoiding and curing ailments and help protect human health. Traditional medicinal plants are widely used for healthcare and cure of ailments for 2000 years by most of the world. It is also widely accepted because of the perception that they are safe and have a long history in folk medicine for treating ailments from ancient times [3]. Plants have improved sophisticated defense mechanisms to keep alive in their ecosystems and are therefore a large source of pharmaceutical compounds. The most interesting application area for medicinal plant extracts is inhibition of growth and a decrease in the number of pathogens. Only 1% of 500,000 plant species found globally have been studied phytochemically, mainly in terms of antimicrobial and antibiofilm activities. Various plant extracts containing 54 plants and common food products, 6 Florida plant extracts against *P*. *aeruginosa*, 168 plant extracts against *S*. *aureus*, 13,000 fractions of 167 plant genera, 6 herbal plants, and a few grapefruit derived flavonoids against *Escherichia coli* strains have been studied in relation to the control of pathogenic biofilms [13]. Furthermore, antibacterial properties and mainly biofilm prevention research from plant-based natural products are supported by the fact that phytochemicals are less toxic to the environment [7]. Some of the plants with antimicrobial effects containing saponin compounds had possible antibiofilm activities against isolated nosocomial bacteria; it can be an alternative to control microbial biofilm formation or can be used as a model for research of novel drugs [3]. Considering the above, this review focused on the biofilm-forming microorganisms, attempts to and control against these infections, and the antibiofilm potency of saponin-containing plants having antimicrobial properties.

## 2. Secondary Metabolites from Medicinal Plants: Saponins

Saponins (Latin word “*sapo*”) have a distinctive foaming characteristic when diluted in aqueous solution and include steroidal or triterpenoid aglycones attached to one or more sugar units. They have significant biological characteristics such as cytotoxic, hemolytic, molluscicidal, anti-inflammatory, antifungal, antiyeast, antibacterial, and antiviral activities, and chemical structures that present natural nonionic detergent properties. Saponins can be divided into two groups, depending on the nature of the aglycone skeletons: steroidal and triterpenoid structures. The biological characteristics of saponins are based on the aglycone structure and the number of sugar chains covered. Their therapeutic potential against eukaryotic cells is linked with cell membrane permeabilizing characteristics complexing with cholesterol [14].

Saponins can impair the permeability of the bacterial outer membrane. About 90% of the surface of the Gram-negative bacteria cell wall outer membranes that do not contain natural cholesterol are covered with lipopolysaccharide (LPS). Saponins can interact with the lipid A part of Proteus LPSs, thereby increasing the permeability of the bacterial cell wall because of their detergent-like properties. Theoretically, this activity can facilitate influx of antibiotics through the bacterial cell wall membrane. Lipid A-saponin complexes can assist the intake of antibiotics (colistin and ampicillin) into naturally resistant bacterial cells. Previously, in the presence of 15 *µ*g/mL saponin, colistin or ampicillin has been shown to reduce the number of cells in the laboratory strains of *Proteus mirabilis* S1959 and R45. To eliminate pathogenic Gram-positive and Gram-negative bacterial strains, various types of methods were studied, such as bacteriophage treatment, prevention of bacterial adhesion, and enhancing bacterial cell wall permeability. Therefore, saponin abilities and the concentrations to stimulate hemolytic and cytotoxic effects against eukaryotic cells, to interfere with antibiotics against bacterial cells, and their influences on the growth of bacterial and fungal strains are medically desired condition [14].

## 3. The Antibiofilm Effects of Plants with Antimicrobial Properties Containing Saponin-Type Compounds

### 3.1. *Agave sisalana* Perrine (Asparagaceae)

*Agave sisalana* is native to Southern Mexico and largely cultivated in many countries. Leaf juice is traditionally used in Northern Morocco as a wash for treating skin diseases, and for pulmonary tuberculosis, syphilis, and jaundice. This species was found to have potent antimicrobial activity against several Gram positive, Gram negative, and fungus such as *Staphylococcus aureus*, *E*. *coli*, and *Bacillus cereus* [15]. *Agave* collected from different agricultural fields was to screen for in vitro antibiofilm and antiquorum sensing activities. The activity of the ethanolic extract of *Agave sisalana* on inhibiting biofilm formation was tested using the crystal violet method widely used by microbiologists. The extract has the highest antibiofilm activity against the test microorganism with an 87.5% reduction in biofilm formation. A low extract concentration may be necessary to prevent initial attachment of the biofilm, while a higher concentration will disrupt the preformed biofilm [16].

### 3.2. *Anacardium occidentale* L. (Anacardiaceae)

*Anacardium occidentale* (cashew) is a plant species that spreads in most tropical and subtropical countries and has long been utilized in traditional medicine for treating various infectious ailments. The bark is utilized for asthma, inflammatory diseases, and wound healing treatments, to detoxify a snake bite, and for fevers, a laxative, to get rid of intestinal parasites, and to cure diabetes in Brazil and African countries [5, 17]. In Brazil, physicians prescribed the hydroethanolic extract of stem bark of titled plant as drops for dilution in water before application. Therefore, the antibacterial and antibiofilm activity of the extract at different concentrations was investigated. The concentrations of 122, 61, 30.5, and 15.2 mg/ml in the tests displayed the activity on all planktonic cells (*S*. *aureus* and *S*. *epidermis*), while reduced concentrations influenced several strains. For biofilms, 122, 61, and 30.5 mg/ml concentrations were active against all strains, but this was not observed at reduced concentrations. It was also observed that strains were completely destroyed. Phytochemical analysis performed on the extract showed the presence of compounds such as tannins, saponins, flavonoids, and phenols defined as the major active components in the extract [5]. Saponin concentration was significantly high in cashew bark extract [17].

### 3.3. *Astragalus angulosus* DC. (Fabaceae)

*Astragalus* species have been utilized in folk medicine for a long time. They have been used to promote and strengthen the immune system, for colds, upper respiratory tract infections, and heart diseases, as well as chronic hepatitis and other viral infections, also used as an adjunct therapy for cancer. The aqueous and ethanolic extracts of all parts of *Astragalus angulosus*, a Lebanese endemic species, were tested and evaluated to find the antibacterial and antibiofilm activities against five bacterial strains: Gram-positive bacterial strains such as *S*. *epidermidis*, *S*. *aureus*, and *Enterococcus faecalis* and Gram-negative strains such as *E*. *coli* and *P*. *aeruginosa*. Saponins, coumarins, and flavonoids were found in all extracts and parts of the plant but in variable quantities based on diverse fractions. The ethanolic extract of whole plant had the highest bacteriostatic effect at 12.78 mg/ml concentration and was the most sophisticated that exerts its impact against 3 different strains. Another extracts also had an efficacy, however at higher concentrations and each against a single strain. Concerning the antibiofilm activity, most of the extracts were able to exterminate > 50% of *S*. *epidermidis* preformed biofilm, where the highest activity was achieved with the fraction of flower extracted in water and 67.7% biofilm eradication at 0.2 mg/ml was achieved [6].

### 3.4. *Atriplex tatarica* L. (Amaranthaceae)

**Figure 1:**
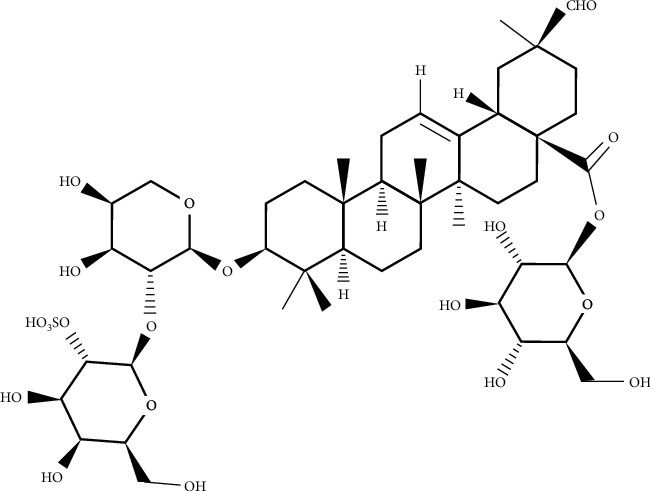
Chemical structure of atriplexogenin I.

The genus *Atriplex* is a plant that grows in the world's largest continuously arid region. Many plants from the genus are known to be edible and widely used in folk medicine for various ailments. These species have antimicrobial activity and active bactericidal components. Chemical research of the *Atriplex tatarica* consumed as food in several European countries including Turkey displayed flavonoids, saponins, and alkaloids as the major secondary metabolites. Their antibacterial and antibiofilm activities against *P*. *aeruginosa* were evaluated by the microdilution method. Patuletin 3-*O*-*β*-D-apiofuranosyl-(1‴⟶2″)-*β*-D-glucopyranoside as a flavonoid compound has significant antibacterial activity, while atriplexogenin I (Figure 1) as a triterpenoid saponin derivative has the strongest antibiofilm agent. The tested compounds displayed antibiofilm activity against *P*. *aeruginosa* at sublethal concentrations at 0.5, 0.25, and 0.125 of MIC values. At the concentration of 0.5 MIC, all compounds decrease biofilm formation in the range of 20.07–58.06%, while some presented activity at the lowest concentration (0.125 MIC). The most promising agent on antibiofilm activity was atriplexogenin I (56.63–7.17%) at all concentrations, while patuletin 3-*O*-*β*-D-apiofuranosyl-(1‴⟶2″)-*β*-D-glucopyranoside (58.06 and 12.19%) and atriplexogenin III had potential at 0.5 and 0.25 of MIC values. Streptomycin and ampicillin reduced the biofilm at 52.46–89.31% and 58.94–85.24% values in all sub-MIC tested, respectively. The outcomes indicated that especially triterpenoid derivatives presented significant biofilm inhibition of *P*. *aeruginosa* [8].

### 3.5. *Bacopa monnieri* (L.) Wettst. (Plantaginaceae)

**Figure 2:**
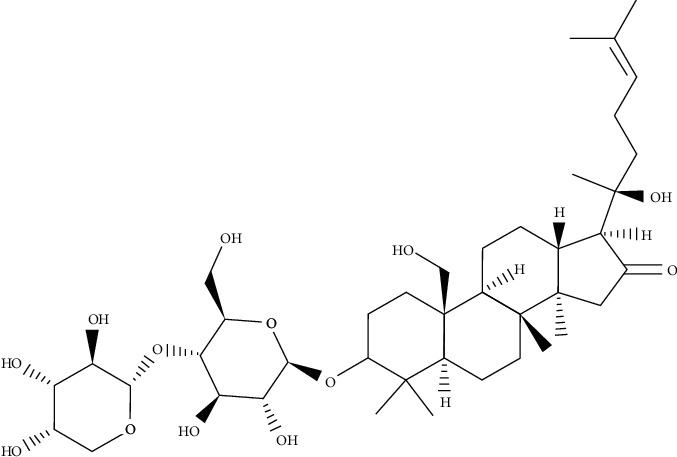
Chemical structure of Bacoside A.

*Bacopa monnieri* is a widespread plant growing in tropical countries and used as a medicinal plant for centuries as a memory enhancing, nervine tonic, hepatoprotective, and cardiotonic agent [18]. The extract of the plant has shown various therapeutic effects against anxiety, gastrointestinal diseases, skin disorders, epilepsy, pyrexia, and analgesia. Bacoside A (Figure 2), a saponin compound, is recognized as the major effective component of *B*. *monnieri*, and demonstrated its potential as an antimicrobial and antibiofilm agent against two opportunistic pathogenic bacteria, *S*. *aureus* and *P*. *aeruginosa*. Bacoside A can be regarded as an effective antimicrobial agent at <400 *µ*g/mL MIC value. Biofilm quantification by crystal violet assay, Bacoside A, could eliminate 88–93% (200 *µ*g/mL) bacterial biofilm of selected bacterial strains from the biofilm substratum. Bacoside A displayed obvious evidence of biofilm dispersion due to EPS loss. Previous studies also proposed that the inhibition of EPS production leads to the elimination of bacterial biofilm. The high eradication of the bacterial biofilm after the treatment with Bacoside A was presented by SEM images qualitatively. MTT analysis also determined the percentage of mortality of bacterial cells in a treated biofilm (200 *µ*g/mL) [19, 20].

### 3.6. *Bellis perennis* L. (Asteraceae)

*Bellis perennis* is a plant native to almost all of Europe. It has been used in folk medicine for the treatment of various diseases, e.g., rheumatism, and as an expectorant [21]. The aqueous extract of the aerial parts of *Bellis perennis* that has antimicrobial, antibiofilm, and quorum-sensing inhibitory activities widely used as edible vegetables in Southwest Anatolia and contains triterpenoid saponins was studied. Antimicrobial activity was assessed against 15 bacterial strains and *Candida albicans* using the disk diffusion and broth microdilution assays. Antimicrobial and antibiofilm activity experiments demonstrated that *B*. *perennis* aqueous extract has moderate antimicrobial and antibiofilm activities against *S*. *epidermidis* MU30, *P*. *aeruginosa* ATCC 27853, and *P*. *fluorescens* MU 181 at a concentration of 10 mg/ml. The 100 mg/ml aqueous extract of *B*. *perennis* presented promising antiquorum-sensing activity on *Chromobacterium violaceum* CV026 with an inhibition zone of 10 mm. Aqueous extracts of *B*. *perennis* blocked swarming by 9.5%. The results show that *B*. *perennis* may be an alternative resource for exploring beneficial ingredients in the fight against bacterial infections [22].

### 3.7. *Calendula officinalis* L. (Asteraceae)

**Figure 3:**
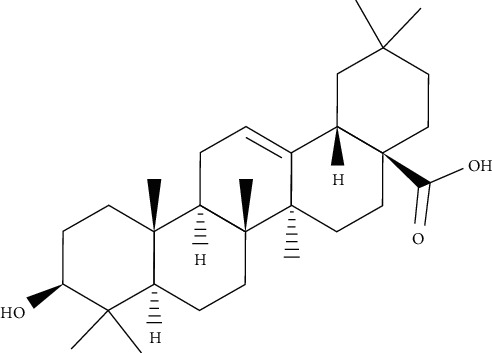
Chemical structure of oleanolic acid.

*Calendula officinalis* has traditionally been used to treat oral and pharyngeal mucositis, wounds, and burns. It is a cleansing and detoxifying plant, and the plant infusion cures chronic infections [23]. Its aqueous extract was utilized to treat skin diseases and pain and as a bactericide, and antiseptic and anti-inflammatory agents exhibited significant antibacterial effects against all pathogenic bacterial strains at 100 *µ*g/ml concentration, especially when compared to cephotan antibiotic. *Shigella sonnei* was more susceptible to the extract than other bacteria with the highest inhibition zone (23 mm) at 100 *µ*g/ml concentration. The concentration of 25 *µ*g/ml of aqueous extract showed poor effect against bacterial strains, except for *S*. *sonnei*. It was stated that *C*. *officinalis* flowers' aqueous extract showed preferable antibacterial activity in comparison with other extracts from different parts (leaves, roots, and stems). They showed also remarkable antibacterial activities against *E*. *coli*, *P*. *aeruginosa*, *Enterococcus* sp., and *Staphylococcus* sp. Considering as potential antibacterial and therapeutic agents, oleanolic acid (Figure 3) and its glycosides from *C*. *officinalis* can be proposed. Bacterial strains (*Salmonella*, *Shigella dysenteriae*, *S*. *flexneri*, *S*. *sonnei*, and *E*. *coli*) had the ability to adhere to different degrees on the smooth surface of glass tubes. *Salmonella* gave adherent growth in large amounts while other bacterial isolates (*S*. *sonnei*, *S*. *flexneri*, and *S*. *dysenteriae*) gave adherent growth in a small amount. Aqueous extracts of *C*. *officinalis* have been found to reduce the adhesive growth of bacteria on glass tubes. Conversely, the extract of *C*. *officinalis* blocked the bacterial adhesion on the polystyrene surface and as a result caused the separation of biofilms, which caused a decrease in the absorbance values of the biofilms. These activities, recorded for *C*. *officinalis* flower extract, let them to be listed as potential antibiofilms and antibacterial natural agents. This may suggest that they are used as therapeutic agents to treat biofilm-related infections induced by enteric pathogens. The mechanism of the effect on the biofilm may differ for each plant extract [1].

### 3.8. *Centella asiatica* (L.) Urb. (Apiaceae), *Cinnamomum zeylanicum* Blume (Lauraceae), and *Mentha spicata* L. (Lamiaceae)

*Centella asiatica* is native to Southeast Asian countries and has been traditionally used for wound healing, eczema, and burn and scar treatment and for stress and anxiety, as well as to treat periodontal disease for a long time. The inner bark of the genus *Cinnamomum* is a common herbal drug used as a spice in various countries by different cultures around the world, besides, for inflammatory diseases and diabetes, against infections (bacteria and fungi). Spearmint is native to Northern England and is widely grown in tropical and temperate regions such as Europe, South Africa, and Brazil. It is used for fevers, headache, digestive disorders, bronchitis, ulcerative colitis, and liver complaints in folk medicine. The biological activities of these species include antimicrobial and anti-inflammatory effects. The extracts of medicinal plants, *Centella asiatica*, *Mentha spicata*, and *Cinnamomum zeylanicum*, were screened against multidrug resistant *P*. *aeruginosa* strains for their antibacterial and antibiofilm properties. The antibiofilm activity test of methanol extracts proposed that *C*. *zeylanicum* and *M*. *spicata* extracts are potent antibiofilm agents whilst the *C*. *asiatica* extract is a moderate antibiofilm agent. Ethylacetate extracts of *C*. *zeylanicum* and *M*. *spicata* displayed strong antibiofilm activity. For the evaluation of the compounds responsible for antibacterial and antibiofilm activities of the selected plants, the phytochemical analysis was done according to ethnobotanical data and it was suggested that *C*. *asiatica* leaves, *C*. *zeylanicum* barks, and *M*. *spicata* leaves, stems, and flowers contain saponins and they are responsible for the activity [24].

### 3.9. *Cyclamen coum* Mill. and *C. hederifolium* Aiton (Primulaceae)

*Cyclamen coum* known as a medicinal plant spreads in the forests of the Golestan area of Iran. It is known that many *Cyclamen* species are used for hemorrhoids, eczema, and wound healing. There is also information that their tubers are used as a sedative, anthelmintic, and laxative, as well as expelling digestive system worms. Tubers of the plant include large quantities of saponins (156 *μ*/mL). Increased levels of saponins can be obtained using different solvents and especially collected from the *n*-butanol phase. The effects of the combination of *C*. *coum n*-butanol extract and ciprofloxacin on the prevention of biofilm formation of *P*. *aeruginosa* were evaluated. Antibiotic (alone, at 6 × MIC) or *n*-butanol extract of *C*. *coum* (alone, at 55 + 0.3 *μ*g/mL) remarkably impaired *P*. *aeruginosa* biofilm formation, but their combinations blocked more prominent biofilm formation (both antibiotic and *n*-butanol extracts of *C*. *coum* concentrations decreased at 3 × MIC and 38 *µ*g/mL, respectively). The *n*-butanol extract of *C*. *coum* had a synergistic effect against *P*. *aeruginosa* biofilms in combination with ciprofloxacin. It is logical to stand that the extract that contains saponins may resensitize antibiotic-resistant bacteria by preventing cell-cell communication (quorum detection) [25].

*C*. *hederifolium* Aiton is a plant with a common Mediterranean element in Çatalca and Kocaeli in Turkey. Its tubers are pounded and placed in a certain amount of water, and then the filtered water is given to the tobacco plantation and used as a pesticide to kill harmful insects. This effect is due to the fact that the saponins in its content are used to defend themselves against harmful factors that may come from the environment. In a study conducted with *C*. *hederifolium*, antibiofilm activity against *Staphylococcus aureus* was tested by microdilution method, and MIC values (≤32 *μ*g/ml) were determined [26].

### 3.10. *Dioscorea panthaica* Prain et Burk. (Dioscoreaceae)

**Figure 4:**
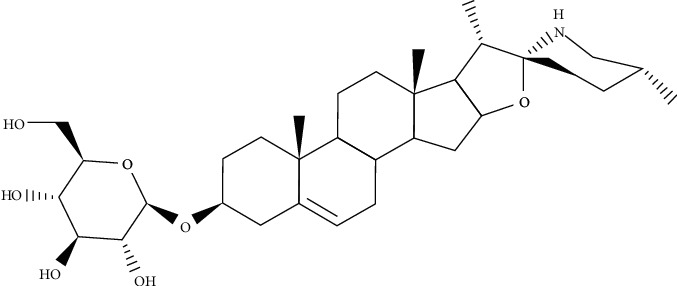
Chemical structure of solasodin-3-*O*-D-glucopyranoside.

Traditionally, *Dioscorea panthaica* has ameliorated the symptoms of cardiovascular diseases. This medicinal endemic plant can also be utilized as a topical medicine in the treatment of infectious ailments induced by microbial pathogens such as *Lymphatic tuberculosis* and *Bacillus anthracis*. The saponin fraction from *D*. *panthaica* dried rhizomes (Huangshanyao saponin extract, HSE) against *C*. *albicans* was investigated for its antifungal effects. HSE blocks the planktonic growth, biofilm formation, and improvement of *C*. *albicans*. At 16–64 *μ*g/mL, HSE reduced the viability of adherent cells on the polystyrene surfaces, the transition from yeast to filamentous growth, and generation of phospholipase and cell membrane disruption in planktonic cells. The administration of 64 *μ*g/mL dose of HSE blocked 80% of the adhesion in comparison with drug-free control groups. It was determined that inhibitory effects against extracellular exopolysaccharide production in preformed biofilms could be provided by 64–256 *μ*g/mL of HSE. Hyphae behave as a critical part of the infection and biofilm formation. Throughout mucosal-related infections, hyphae attack epithelial and endothelial cells, thereby causing destruction in which hydrolytic enzymes such as phospholipase act a significant role. The presence of hyphae builds the biofilm more compatible and assists *C*. *albicans* cells to harm epithelial cells and diffuse deep into tissues. Therefore, there comes the idea that reducing the growth of hyphae could possibly alleviate the harm to the hosts. HSE and the pure compounds, solasodin-3-*O*-D-glucopyranoside (Figure 4), and purpurin were able to prevent the growth of *C*. *albicans* [27].

### 3.11. *Erica manipuliflora* Salisb. (Ericaceae)

The genus *Erica* L. is symbolized by more than 700 species in the world, among these species, *Erica manipuliflora* is commonly found in coastal areas in Turkey. The herbal teas of *E*. *arborea* and *E*. *manipuliflora* prepared from their aerial parts are common in Turkey as a diuretic and astringent and utilized to treat urinary tract infections. The *n*-butanol extract of *E*. *manipuliflora* has been reported to have significant activity against some marine biofilm bacteria (*Pseudoalteromonas marina*, *P*. *haloplanktis*, *P*. *elyakovii*, *P*. *porphyrae*, *P*. *agarivorans*, *Alteromonas genoviensis*, *Vibrio lentus*, and *Exiguobacterium homiense*). All biofilms investigated in the study were generally resistant to both antibiotics (vancomycin and tobramycin), and *E*. *homiense* and *A*. *genoviensis* were more sensitive. It has been suggested that the *n*-butanol fractions rich in flavonoids and triterpenoid saponins of the plants used in the study may be sources for the invention of new antibiofilm agents from plant sources [13].

### 3.12. *Glycyrrhiza glabra* L. (Fabaceae)

**Figure 5:**
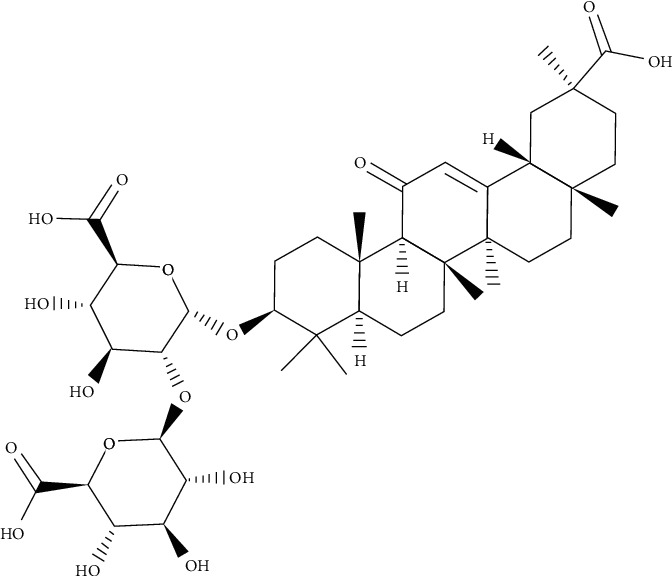
Chemical structure of glycyrrhizin.

*Glycyrrhiza glabra* root, grown in Eastern Anatolia, is one of the most widely used herbal remedies and a significant source of confectionery. The pharmacological characteristics linked with *G*. *glabra* are well documented. In accordance with the World Health Organization, it is used as a sedative to treat sore throats and as an expectorant for cough and bronchial flu. In addition, it plays a crucial role in the prophylaxis and the therapy of gastric and duodenal ulcers as an anti-inflammatory agent, as well as it has been used for more than 20 years as a treatment for chronic hepatitis. *G*. *glabra* extract decreases surface tension and shows antimicrobial activity against both Gram-positive and Gram-negative bacteria. The antimicrobial effects of *G*. *glabra* roots and leaves, especially owing to glycyrrhizin (Figure 5), have been recorded. Glycyrrhizin interacts with membrane lipids and disrupts cell membrane integrity that causes cells to abandon intracellular organelles. In addition, *G*. *glabra* root extract including 7–7.5% glycyrrhizin was found to be advantageous in terms of cytotoxicity. Therefore, *G*. *glabra* roots have a crucial effect on biofilm which could be caused by inhibition of cell integrity, causing wall and membrane damage and leakage. The ethanolic extract of *G*. *glabra* roots has exceptional prevention effects against the planktonic growth of *S*. *pyogenes* and is the most efficacious bactericidal agent that killed 99.99% of the initial bacterial load within 3 h exposure to the 2 × MIC. Although *G*. *glabra* extract has significant antibiofilm activity, its effectiveness is lower than NaOCl and cetrimide which are the most effective agents against *E*. *faecalis* biofilms on dentine discs [28, 29].

### 3.13. *Hibiscus tiliaceus* L. (Malvaceae)

The plant parts are utilized for cuts, tuberculosis, and conjunctivitis in the Solomon Islands. In New Guinea, the bark is utilized as cough remedy and used for tuberculosis. The leaves are utilized to treat cough, sore throat, and open wounds. A formulation made from leaves, roots, and bark is given for fever. The leaves boiled with sugar in Java are used in the treatment of cough and bronchitis. The leaves and bark are utilized in traditional Chinese medicine to treat cough and bronchitis. In Bangladesh, leaves are used in traditional medicine for fever, cough, and dry throat, while flowers are used for bronchitis, ear infections, dysentery, and chest congestion. Extracts and fractions of different parts of *Hibiscus tiliaceus* exhibited antibacterial and antibiofilm effects against *P*. *aeruginosa*. The chemical components found in *H*. *tiliaceus* have been found to vary between plant parts, and flavonoids, phenolics, steroids, and terpenoids were the main compounds that could contribute to these activities. Saponins appear to be more involved in the fruit parts of the plant. Fruits were extracted with methanol and fractionated with chloroform, ethylacetate, and methanol. While the chloroform fraction, which contains mostly nonpolar structures, was most effective in terms of antibacterial activity, all extracts and fractions have the capacity to remarkably block the biofilm formation in terms of antibiofilm activity [7].

### 3.14. *Medicago sativa* L. (Fabaceae) and *Saponaria officinalis* L. (Caryophyllaceae)

**Figure 6:**
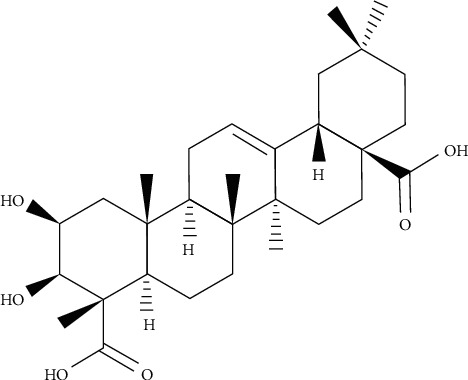
Chemical structure of medicagenic acid.

**Figure 7:**
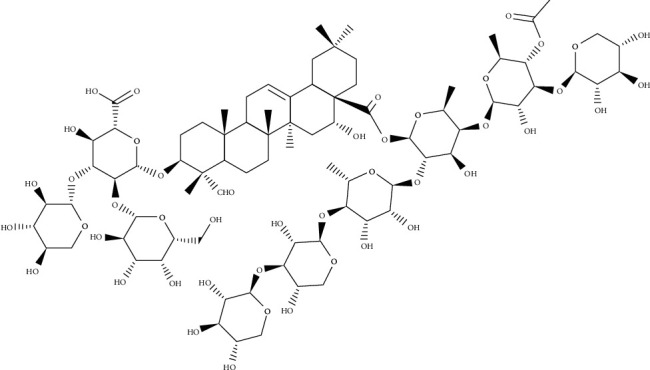
Chemical structure of saponariosides A.

**Figure 8:**
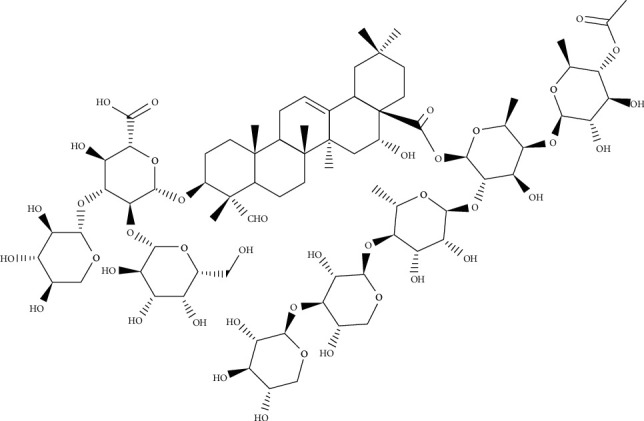
Chemical structure of saponariosides B.

*Medicago sativa* is a plant known since ancient times for animal breeding and nutrition. From historical documents, it is more likely to be of eastern, especially Iranian origin. The ancient Persians' acquaintance with this herb and their use are reflected in the sources of their neighbors, from the heart of the Persian kingdom eastward to China and westward, from Mesopotamia to Europe in antiquity. In folk medicine, the herbal drug is used to treat diabetes and malfunctioning of the thyroid gland and cure for digestive systems [30]. Pharmacological activities of *M*. *sativa* saponins include hemolytic activity, cytotoxic properties, nematocidal and insecticidal activities, and an antimicrobial effect mainly against Gram-positive bacteria and some fungi, plants, and some human pathogens. *Saponaria officinalis* is a common perennial and native plant that extends throughout Europe and Asia. The uses of saponins obtained from *S*. *officinalis* in traditional medicine are mainly as an expectorant during the course of upper respiratory tract infections or for the treatment of skin and rheumatic lesions [10]. Medicagenic acid glycopyranosides (containing medicagenic acid as aglycones, Figure 6) are the major components of *M*. *sativa* extracts [31], additionally, saponariosides A (Figure 7) and B (Figure 8) are the main compounds of *S*. *officinalis* [32]. Since few synthetic antimycotic agents have been identified, including polyenes, azoles, echinocandins, allylamines, and 5-flucytosine, one of the lesser-known but promising agents, saponins which are surface-active phytochemicals, in this regard has been investigated. The antifungal properties against *C*. *albicans* with regard to antibiofilm activity and their synergistic effect with classic antimycotic of the saponins obtained from *Medicago sativa* and *Saponaria officinalis* have been studied [10]. The ability of saponin-rich extracts to inhibit *Candida* germ tube formation, which corresponds to limiting hyphal growth, is so crucial. This activity can prevent the development of invasive mycosis and fungal biofilm formation in the early stages of infection. Moreover, these extracts have been shown to have a significant effect on the reduction of *C*. *albicans* adhesion and biofilm generation and the elimination of mature *Candida* biofilm [33]. It was shown a significant antifungal potential of saponins obtained from the aerial parts and roots of *M*. *sativa* and the roots of *S*. *officinalis* alone or with antimycotic synergies. The ability of saponin-rich extracts to negatively affect *C*. *albicans* virulence factors such as germ tube formation, hyphal growth, adhesion, and biofilm formation has been indicated. These qualities of *Candida* cells play a role as new potential drug targets, and therefore, the properties of saponins seem very hopeful in the context of their potential medical application [10].

### 3.15. *Scrophularia ningpoensis* Hemsl (Scrophulariaceae)

The genus *Scrophularia* mainly occurs through mountainous regions is one of the large genera of the Scrophulariaceae and used as a heart and circulatory stimulant, and diuretic. Besides, the other traditional uses of this genus include antipyretic, febrifuge, antibacterial, antierythema, anticonstipation, and antifurunculosis properties, ulcerous stomatitis, and tonsillitis treatment, as well as anti-infections' treatment in different types of disorders. The antibacterial activity of *Scrophularia ningpoensis* Hemsl extract on biofilm formation of *Klebsiella pneumonia* was investigated. The biofilm-forming capacity was conducted using the colony-forming unit test. The extract displayed considerable antibiofilm activity. Inhibition of bacterial biofilm formation was presented at the concentration of 0.75 mg/ml against *K*. *pneumoniae* with no signs of cytotoxicity in L929 [34].

### 3.16. *Solidago virgaurea* L. (Asteraceae)

*Solidago virgaurea* is a medicinal plant widely used in Europe and other parts of the world and is known among the most researched species in its genus. The aerial parts of the plant have long been used for urinary tract ailments and as an anti-inflammatory agent in traditional medicine of different peoples [35]. In the related study, it was purposed to protect oral bacteria in healthy microflora, natural competitors of *C*. *albicans*, to improve a dry mouth-specific mouthwash and maintain oral mucosal hydration. Based on previous studies and the following effects of *Solidago virgaurea* saponins such as antimicrobial properties, as well as used as detergent, and hemolytic agents owing to their ability to connect membrane sterols, the antibacterial or antifungal activities of *Solidago* saponins have been studied. However, it should be noted that although saponins may interfere with membrane sterols of plants, viruses, and fungi, saponins may not exhibit significant antibacterial activity because most bacteria lack membrane sterols. Therefore, in the study, it was shown that *S*. *virgaurea* aqueous extracts do not have antibacterial or antifungal activity; that is, they do not inhibit bacterial growth. On the other hand, in response to the host environment, *C*. *albicans* yeast-hyphal transition is considerable for virulence. Thus, the second purpose of this study was to block *C*. *albicans* yeast-hyphal transition using *Solidago* extracts, and such an effect was observed in experiments. The extract strongly inhibited biofilm formation and decreased preformed biofilms. The percentages of reduction observed were from 95.86 to 99.46% in biofilm formation and from 76.26 to 92.37% in preformed biofilm formation. As a result, it has been shown that the aqueous extract of *S*. *virgaurea* creates an unfavorable environment for *C*. *albicans* and inhibits the yeast-hyphal transition without killing the yeast form and oral endogenous bacteria [36].

### 3.17. *Terminalia fagifolia* Mart. (Combretaceae)

*Terminalia* genus consists of about 200 species widely used in folk medicine. It is located in the Brazilian Cerrado and is popularly known as “*capitão*, *capitão-do-cerrado*, *capitão-do-campo*, and *mirindiba*.” All parts of *Terminalia* species are mostly given orally as decoctions or macerations to treat a wide variety of infectious diseases such as dysentery, diarrhea, cough, abdominal pain, chest pain, fever, eye infections, and respiratory infections. These species are also used externally as an antirheumatic ointment or poultice, or to heal skin infections such as acne, wound healing, and skin ulcers, and to treat itchy skin. Rodrigues de Araujo et al. [37] searched the pharmacological properties and antibacterial, antibiofilm, and cytotoxic effects of ethanol extract and its fractions of *Terminalia fagifolia* stem barks. The ethanolic extract was suspended in a mixture of H_2_O/MeOH and partitioned with ethyl acetate. The organic phase was concentrated, then suspended in MeOH/H_2_O. Then, it was provided aqueous and hydroalcoholic fractions. Serial dilutions (concentrations were tested at between 12.5 *µ*g/ml and 400 *µ*g/ml) of ethanol extract and its fractions were prepared in the MIC test. Concentrations in antibiofilm activity were tested by diluting at 1/2, 1/4, and 1/8 ratios. Bacteria synthesize and release extracellular polysaccharides so that biofilm-forming bacteria adhere to surfaces. This structure not only helps with adhesion but also acts as a defense barrier. Hydrophobic bonds such as the electric charge and hydrophobicity of the surface and Van der Waals interactions are important between the organism and the material to which the organism will adhere. Extracts and fractions may have inhibited biofilm formation, leading to little-known metabolic changes. These possible changes include a reduction in the production and secretion of exopolysaccharides and a change in the electrical charge and/or hydrophobicity of the bacterial membrane. However, more specific protocols are needed to confirm the role of these fractions. The results thus obtained showed that the ethanol extracts and fractions had high antibacterial activity and that 80% of biofilm formation was inhibited for some strains. It is suggested that the presence of saponins with antimicrobial potential in the aqueous and hydroalcoholic fractions can be responsible for the activity [37].

### 3.18. *Trifolium* L. Species (Fabaceae)

**Figure 9:**
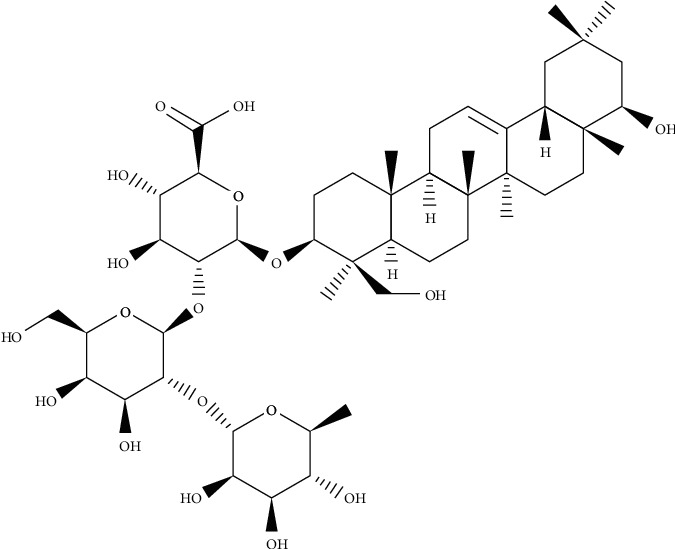
Chemical structure of soyasaponin Bb.

The genus *Trifolium* L., one of the largest genera of the Fabaceae family, attracts attention for their expectorant, analgesic, antiseptic, and antirheumatic properties, as well as containing secondary metabolites, especially saponins and flavonoids [38]. The production of saponins by plants is a remarkable part of their defense against pathogens and herbivores; however, it is well known that they have a much wider range of characteristics such as antimicrobial, hemolytic, anti-inflammatory, cytotoxic, and antitumor activity. Important and well-known virulence factors of *Candida* cells are hydrolytic enzymes such as proteases, lipases, and phospholipases. This plays a role in nutrition, adhesion to host cells, and tissue destruction. The enzymatic activity of yeasts pretreated with saponin-rich (80–98%) fractions isolated from extracts of the aerial parts of *Trifolium alexandrinum*, *T*. *incarnatum*, and *T*. *resupinatum* var. *Resupinatum* was tested. Two triterpenoid glycosides, soyasaponin Bb (soyasaponin I, Figure 9) and soyasaponin *β*b (soyasaponin I conjugated at the 22-position with DDMP) were previously characterized in several clover seeds and found major saponins in the three types of *Trifolium* seeds tested. Treatment of this strain with saponin fractions at 0.5 mg/mL disclosed a statistically significant reduction in the release of certain enzymes, including acid and alkaline phosphatase, naphthyl-AS-BI-phosphohydrolase, and N-acetyl-*β*-glucosaminidase. The production of other enzymes has also been somewhat affected [39].

### 3.19. *Quillaja saponaria* Molina (Quillajaceae) and *Yucca schidigera* Roezl ex Ortgies (Asparagaceae)

*Quillaja saponaria* bark has been used since time immemorial by the Mapuche people, the main ethnic group of South-Central Chile to treat toothache and respiratory infections. This species is well known for its triterpene saponin content. More or less pure saponins and their specific fractions are widely used as vaccine adjuvants. Triterpenic saponins and their aglycones have been displayed to have anti-inflammatory, antiallergic, antiviral, antifungal, and cytotoxic properties [40]. *Yucca schidigera* is a medicinal plant native to Mexico and one of the major commercial sources of steroidal saponins. The main application of *Yucca* products is in animal nutrition, in particular as a feed additive. According to folk medicine, *Yucca* extracts have antiarthritic and anti-inflammatory effects [41]. The antibacterial effects of water extracts from *Yucca schidigera* and *Quillaja saponaria* containing saponins in the range of 12% to 90% were characterized. This study discovered the capacity of saponin extracts to block the adhesion and invasion of HeLa cells cultured by *E*. *coli*, *Yersinia enterocolitica*, *Listeria monocytogenes*, *Salmonella enterica* serovar Typhimurium, *Shigella flexneri*, and *Vibrio cholerae*. The presence of *Y*. *schidigera* extract during infection had the strongest antibacterial effect. Two possible mechanisms of antimicrobial activity of saponin extracts have been investigated. One of them is that some saponin molecules have an affinity for cell membrane cholesterol. In this study, it is presented that pretreatment of cells with *Y*. *schidigera* or *Q*. *saponaria* extracts can modulate cellular membrane cholesterol levels. It was also studied whether saponin extracts could modulate the activity of Ca^2+^ ion channels. The outcomes indicated that the treatment of cells with each saponin extracts, separately, for 6 or 24 hours does not produce a change in the intracellular concentration of Ca^2+^ ions. Consequently, the protective effect of saponin extracts may be owing to modulation of plasma membrane cholesterol, which leads to impaired cell membrane organization. Each of the extracts contains a mixture of individual saponins, each with a slightly different saponin structure. The extract of *Q*. *saponaria* contains saponins with triterpenoid aglycone skeleton, while *Y*. *schidigera* extract contains a saponin mixture with a steroidal aglycone skeleton. Each saponin in the *Q*. *saponaria* extract contains an aglycone skeleton known as quillaic acid to which branched oligosaccharides are attached. Oligosaccharides usually bind to positions C-3 and C-28 on the aglycone skeleton [42].

### 3.20. *Ulmus rubra* Muhl. (Ulmaceae)

**Figure 10:**
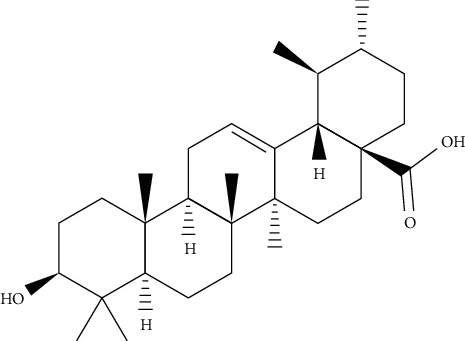
Chemical structures of ursolic acid.

**Figure 11:**
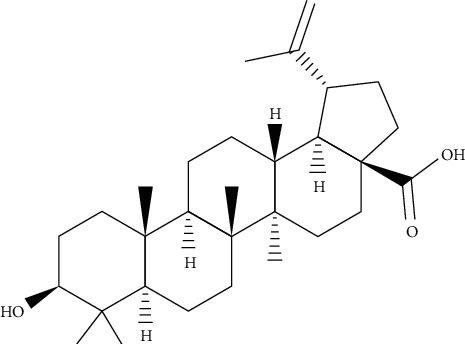
Chemical structures of betulinic acid.

*Ulmus rubra* inner bark is registered to be known traditional healers in Canada for controlling streptococcal pharyngitis. Biofilm development is an important mechanism involved in *S*. *pyogenes* virulence during pharyngitis infections, providing superior survival and protection from host defense mechanisms, antibiotics, and other environmental fluctuations. Therefore, the multiple anti-*Streptococcus pyogenes* attributes involving planktonic growth inhibition, bactericidal effect, morphological disruption in cell wall/membrane, and biofilm inhibition of *U*. *rubra* inner bark extract were assessed. The ethanol extract of *Ulmus rubra* has significant inhibition effects against the planktonic growth of *S*. *pyogenes*. The effects of subinhibitory concentrations of *U*. *rubra* inner bark ethanol extract on biofilm formation over 72 h incubation of three *S*. *pyogenes* strains were quantified by MTT staining. The extract displayed inhibitory activity on biofilm formation ranging from 62.5 to 125 *μ*g/mL. The phytochemical analysis of the extract allowed the isolation of saponins, such as oleanolic acid (Figure 4), ursolic acid (Figure 10), and betulinic acid (Figure 11) that are responsible for the activity [29].

## 4. Antibiofilm Effects of Natural Products

### 4.1. Ankaferd Blood Stopper (ABS)

Ankaferd Blood Stopper (ABS) is a natural product consisting of *Thymus vulgaris*, *Urtica dioica*, *Alpinia officinarum*, *G*. *glabra*, and *Vitis vinifera* and does not contain inorganic or synthetic additives. The plants included in ABS have some effects on blood cells, endothelium, cell proliferation, vascular dynamics, angiogenesis, apoptosis, inflammation, or cell mediators. This product is produced by a registered Turkish company and licensed for use for dental and external and in major or minor bleeding after surgery. The main action mechanism for ABS is the formation of an encapsulated protein network that represents focal points for essential erythrocyte aggregation. The antibiofilm activity of ABS against oral streptococci was demonstrated by *in vitro* analysis and also visualized by SEM. ABS inhibited the growth of the biofilm layer of *S*. *aureus*, *S*. *sanguinis*, *S*. *mitis*, *S*. *sobrinus*, and *S*. *parasanguinis* by 94.48%, 87.00%, 86.57%, 82.38%, and 80.17%, respectively. Nevertheless, at the same concentration, ABS inhibited the growth of *S*. *oralis*, *C*. *albicans*, and *S*. *mutans* biofilm by 62.13%, 38.80%, and 7.89%, respectively. The highest antibiofilm activity of ABS with 94.48% was also monitored by SEM against biofilm formation of *S*. *aureus*. After ABS treatment, planktonic *S*. *aureus* cells could be observed, but there was no biofilm formation in the coverslips. When the chemical structures of the plants in this product were examined, it was observed that the licorice root is rich in saponin. Therefore, it is thought that the most important part of the effect is due to the surfactant compounds such as saponins in this product [43].

## 5. Conclusion/Future Perspectives

Treatments that inhibit planktonic bacteria and fungi have little effect on biofilms. Given the prevalence of biofilm infection, the development of antimicrobial therapies should focus on this form of growth. Biofilm models are critical for the discovery of new antimicrobial agents and to investigate their effects. One approach is to reveal agents that disrupt biofilm processes by allowing antimicrobials to attack biofilms when used in combination. The results can be divided into two categories: samples with antibacterial and antibiofilm effects; patterns containing antibiofilm but without antibacterial activities. In general, biofilm formation decreases due to the reduction of living bacteria. There appears to be a linear correlation between the capacity to block biofilm formation and bacterial growth. Compounds with antibiofilm activity but without antibacterial activity are aimed at biofilm formation, possibly by disrupting the extracellular polysaccharides matrix and the quorum-sensing mechanism, or infecting the nutrient resource without affecting bacterial growth. Although all the mentioned plants in our review show antimicrobial and antibiofilm activities, some of them (*Dioscorea panthaica*, *Glycyrrhiza glabra*, *Solidago virgaurea*, *Terminalia fagifolia*, *Quillaja saponaria*, and *Yucca schidigera*) were found to be more effective in terms of antibiofilm activity by disrupting the extracellular polysaccharide matrix.

In order to be the most medically beneficial, new agents should ideally be able to completely destroy a biofilm. To achieve this degree of activity, various strategies can be considered for the design of new drugs. High-throughput models need to be developed to economically test large molecule libraries for antibiofilm activity. Saponins are characterized by wide antimicrobial activities, and they have interesting chemical structures and properties presenting also antioxidant, anti-inflammatory, and antiapoptotic effects. The interactions between the saponin molecule and cell membrane seem complex, and due to the structural diversity of saponin molecules, it is likely that different mechanisms are involved. To fight infection, their hydrophobic components directly touch the phospholipid bilayers of the microbial cell membrane, causing an enhancement in ion permeability, leakage of vital intracellular components, or disruption and inhibition of pathogen enzyme systems and their respiration, and prevention of protein synthesis and assembly. The damage to the cell membrane might be due to the detergent-like activity of saponins. Saponins' antifungal properties are also linked to the capacity of the major components to pass through the thick fungal cell wall and to locate between the fatty acid chains of the lipid bilayers, disrupt the lipid packaging, and change the structure of the cell membrane. Another feature recommends that saponins can be utilized to promote the activity of antifungal drugs that target sterol compounds of the cytoplasmic membrane (polyenes and azoles). The effective synergistic interactions with saponin fractions and triazole-fluconazole against *Candida* strains with different sensibility were proved. Besides, saponins have made susceptible *C*. *albicans* more susceptible, and the resistance of *C*. *glabrata* strain reduced.

It will be fascinating to see how this information is applied to the discovery of antibiofilm drugs as our understanding of biofilm formation, the diseases it contributes to, and the treatment requirements continue to grow.

## Abbreviations

ABS: Ankaferd Blood Stopper
EPS: Extracellular polysaccharide
HSE: Huangshanyao saponin extract
LPS: Lipopolysaccharide
MIC: Minimum inhibitory concentration
MTT: (3-(4,5-Dimethylthiazol-2-yl)-2,5-diphenyltetrazolium bromide
SEM: Scanning electron microscope.
